# A Missense Mutation in IRS1 is Associated with the Development of Early-Onset Type 2 Diabetes

**DOI:** 10.1155/2020/9569126

**Published:** 2020-01-25

**Authors:** Juyi Li, Shan Sun, Xiufang Wang, Yarong Li, Hong Zhu, Hongmei Zhang, Aiping Deng

**Affiliations:** ^1^Department of Pharmacy, The Central Hospital of Wuhan, Tongji Medical College, Huazhong University of Science and Technology, No. 21 Shengli Road, 430021 Wuhan, Hubei, China; ^2^Department of General Practice, The Central Hospital of Wuhan, Tongji Medical College, Huazhong University of Science and Technology, No. 21 Shengli Road, 430021 Wuhan, Hubei, China; ^3^Department of Pain, The Central Hospital of Wuhan, Tongji Medical College, Huazhong University of Science and Technology, No. 21 Shengli Road, 430021 Wuhan, Hubei, China; ^4^Department of Endocrinology, The Central Hospital of Wuhan, Tongji Medical College, Huazhong University of Science and Technology, No. 21 Shengli Road, 430021 Wuhan, Hubei, China

## Abstract

There could be an overlap of monogenic diabetes and early-onset type 2 diabetes mellitus. Precise diagnosis of early-onset diabetes has proven valuable for understanding the mechanism of diabetes and selecting optimal therapy. The majority of maturity onset diabetes of the young (MODY) pathogenic genes in China is still unknown. In this study, a family with suspected MODY was enrolled. Whole-exome sequencing (WES) was used to analyze the variants of the proband. Variants were filtered according to their frequency, location, functional consequences, and bioinformatics software. Candidate pathogenic variants were validated by Sanger sequencing and tested for cosegregation in other members of the family and nonrelated healthy controls. KEGG (Kyoto Encyclopedia of Genes and Genomes) and PPI (protein-protein interaction) analysis were conducted using the DAVID (Database for Annotation, Visualization, and Integrated Discovery) and the STRING online analysis tools for the candidate pathogenic gene. A total of 123291 variants including 105344 SNPs and 17947 InDels were found in WES. A likely pathogenic rare missense heterozygous mutation in diabetes genes (c.2137C > T, p.His713Tyr in *IRS1*) was identified, which was a cosegregate in this family and not in nonrelated healthy controls. The position of the mutation in the aminoacid sequence of the gene is highly conserved among the species. 2 significantly enriched KEGG pathways were identified including bta04930, type II diabetes mellitus (*GCK*, *INS*, *PDX1*, *ABCC8*, and *IRS1*), and bta04910, insulin signaling pathway (*GCK*, *INS*, and *IRS1*). PPI analysis displayed that IRS1 interacts with 3 known pathogenic proteins including INS, KCNJ11, and GCK. We conclude that WES could be an initial option for genetic testing in patients with early-onset diabetes. *IRS1* p.His713Tyr is implicated as a possible pathogenic mutation in monogenic diabetes, which might require further validation, and the precise molecular mechanism underlying the influence of *IRS1* p.His713Tyr on the development of diabetes remains to be determined in the further prospective studies.

## 1. Introduction

There were 415 million people living with diabetes worldwide in 2015, and this number will rise to 642 million in 2040 and about 193 million people still undiagnosed [1]. Diabetes mellitus is classified as type 1 diabetes mellitus (T1DM), type 2 diabetes mellitus (T2DM), gestational diabetes mellitus, and other specific diabetes mellitus [2, 3].

Patients with early-onset diabetes are more likely to have a positive family history of diabetes [4]. However, there could be an overlap between monogenic diabetes belonging to specific disease forms and early-onset diabetes mellitus in those who are diagnosed at an age younger than 30 years and positive parental history of diabetes [5]. Maturity-onset diabetes of the young (MODY) is reported to be the most common form of monogenic diabetes mellitus; therefore, some early-onset diabetes mellitus is probably MODY. There are 14 MODY subtypes having been listed on the Online Mendelian Inheritance in Man (OMIM) database [6]. MODY is estimated to make up approximately 1-2% of all diabetes and is difficult to distinguish T1DM from T2DM [7]. However, It is estimated that more than 80% of MODY are currently undiagnosed or misdiagnosed as T1DM or T2DM [8, 9]. A correct genetic diagnosis of MODY may alter the therapeutic approach: patients with glucokinase (*GCK*) MODY usually do not require pharmacological treatment, and those with either hepatocyte nuclear factor 4-*α* (*HNF4A*) or hepatocyte nuclear factor 4-*α* (*HNF1A*) MODY are very sensitive to sulfonylurea [8]. However, a majority of Chinese MODY people are due to the defection of unknown genes [10]; thus, the screening of pathogenic genes in diabetic families and the appropriate treatment choice will be of great significance, especially in China.

With the development of science and technology, high-throughput next-generation sequencing instruments are emerging and the cost of sequencing is decreasing. Whole-exome sequencing (WES) is capable of investigating all the functional sequence variations in known genes and used in various fields including monogenic diabetes [11]. WES is likely be used for differentiating monogenic diabetes and early-onset diabetes mellitus. However, the clinical utility of WES in the diagnosis of early-onset diabetes has not been well studied. In our study, we performed WES for the genetic diagnosis of monogenic diabetes in a family with early-onset diabetes in China.

## 2. Materials and Methods

### 2.1. Patient

Age at diagnosis was estimated by the patient's recall or review of medical record. Classification and diagnosis of diabetes were in accordance with ADA's latest guidelines [12]. The participants were defined as healthy controls is when fasting blood glucose, 2 hours postprandial blood glucose, and glycated hemoglobin were all within the normal range. This study was approved by the ethics committee of the Central Hospital of Wuhan. Peripheral blood and urine were collected from all participants. Written informed consent was obtained from the participants.

### 2.2. Sample Preparation and Whole-Exome Sequencing

Genomic DNA was extracted from peripheral blood samples using a TIANGEN DNA extraction kit (TIANGEN, Beijing, China). Sample preparation and pretreatment for next generation sequencing were performed using the SureSelect Human All Exon V5 kit (Agilent). Exome sequencing was carried out on an HiSeq2500 system (Illumina).

### 2.3. Genetic Analyses

The sequence reads were mapped and aligned to the Human Reference Genome (UCSC hg19, NCBI build 37). Variants including SNVs and indels were referenced to public databases including ExAC, dbSNP, ESP, 1000 Genomes, and gnomAD. Analysis was performed with preference to variants located in genes published in PubMed and OMIM, which was implicated in maturity-onset diabetes of the young, early-onset diabetes, and diabetes. Analysis focused on nonsynonymous coding variants, frameshift indel variants, and splice site variants; however, nonexonic and synonymous variants were excluded. The effect of the identified missense variants were evaluated using silico prediction tools including SIFT, PolyPhen2, Mutation Taster, Mutation Assessor, FATHMM, GERP_plus, PhyloP100, and PhastCons100 [13, 14].

### 2.4. Sanger Sequencing

Sanger sequencing was carried out to confirm and cosegregate the candidate variants in the proband and the family and in unrelated healthy controls (*n* = 200).

### 2.5. Analysis of Molecular Pathways and Protein-Protein Interaction (PPI)

KEGG (Kyoto Encyclopedia of Genes and Genomes) and PPI analysis were conducted using the DAVID (Database for Annotation, Visualization and Integrated Discovery, https://david-d.ncifcrf.gov/tools.jsp) and the STRING (https://string-db.org/cgi/input.pl?sessionId=bG2XqRnLXDUO&input_page_show_search=on) online analysis tools for *IRS1*.

## 3. Results

### 3.1. Characteristics of the Participants

**Figure 1:**
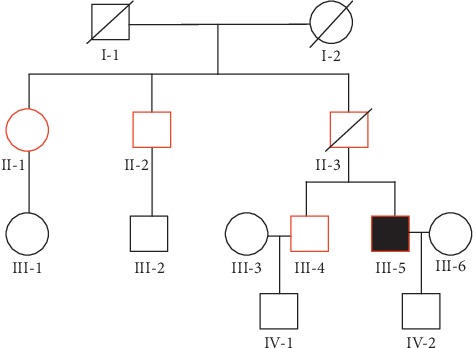
Family tree of the family. The solid black box indicates the proband, box indicates males, circle indicates females, oblique line indicates death, and red indicates diabetes. The proband (**III-5**), his brother (**III-4**), his father (**II-3**), his uncle (**II-2**), and his aunt (**II-1**) were diagnosed with T2DM at the age of 32, 45, 56, 48, and 64, respectively.

Details of the participant characteristics are shown in Table 1. The family tree of the family is shown in Figure 1. Laboratory analysis revealed diabetic autoantibodies were all negative in this family. Oral hypoglycemic drugs are effective in the patient of the family, and diabetic patients presented with impaired islet function. Therefore, the family was initially diagnosed as a type 2 diabetic family.

### 3.2. Mutation Detection

A summary of the whole-exome sequencing data is displayed in Table 2. A total of 123291 variants including 105344 SNPs and 17947 InDels were found in whole-exome sequencing, of which silent mutations, missense variants, nonsense mutations, new SNPs, and new InDels were 11246, 10609, 91, 2026, and 3867, respectively.

### 3.3. Genetic and Bioinformatics Analyses

**Figure 2:**
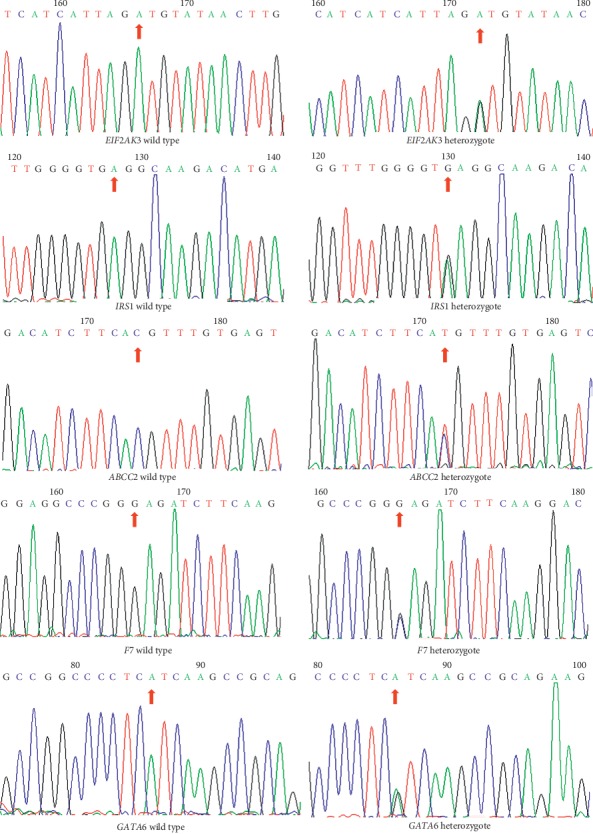
Schematic diagram and sequencing data of the five candidate gene loci. The wild genotype of each candidate gene is listed on the left, and the mutant genotype corresponding to each candidate gene is listed on the right.

Variants were filtered according to their frequency, location, and functional consequences, and the results suggest that the family is not a known MODY type. When the mutation frequency is greater than 0.005 (public databases including KG, ESP, ExAC, and gnomAD) and the nonsense mutation was eliminated and combined with pathogenicity predicted by bioinformatics (SIFT, PolyPhen2_HDIV, PolyPhen2_HVAR, MutationTaster, MutationAssessor, FATHMM, GERP_plus, PhyloP, and PhastCons) and the reported literature on diabetes mellitus, the last five mutation sites were selected (Tables 3 and 4), which had deleterious effect to protein function and strongly related to diabetes; then, the five mutation sites were sequenced and validated in other members of the family (Table 5) and 200 nonrelated healthy controls (Figure 2); the results showed that only a heterozygous missense mutation with predicted damaging effects in *IRS1* was identified in patients with diabetes—chromosome 2, position 227,661,318, c.2137C > T, NM_005544.2, p. His713Tyr, namely, rs1043152329—which is cosegregated with diabetes. Evolutionary conservation analysis also shows that the p.His713Tyr mutation is highly conserved across multiple species (Table 6). In the Genome Aggregation database, the *IRS1* p. His713Tyr mutation has a very low frequency (2/125568, frequency = 0.00002, TOPMED); therefore, this mutation is predicted to be likely pathogenic based on the above criteria.

### 3.4. KEGG and PPI Analysis

**Figure 3:**
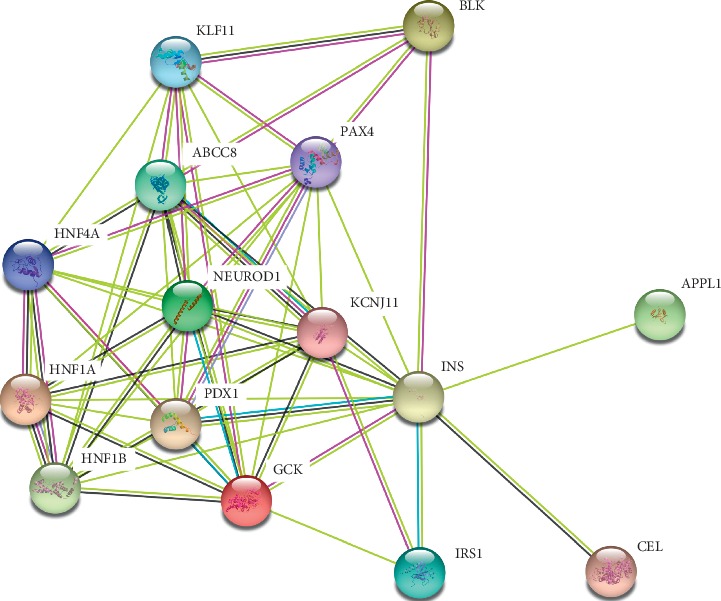
PPI analysis using STRING database. IRS1 interacts with 3 known pathogenic proteins including INS, KCNJ11, and GCK.

To investigate the possible molecular pathways between *IRS1* and 14 known MODY pathogenic genes including *GCK*, *HNF1A*, *PDX1*, *HNF1B*, *NEUROD1*, *KLF11*, *CEL*, *PAX4*, *INS*, *BLK*, *KCNJ11*, *APPL1*, *ABCC8*, and *HNF4A*. Based on KEGG database, 2 significantly enriched KEGG pathways were identified including bta04930: type II diabetes mellitus (*GCK*, *INS*, *PDX1*, *ABCC8* and *IRS1*) and bta04910: insulin signaling pathway (*GCK*, *INS* and *IRS1*) (Table 7). PPI analysis displayed that IRS1 interacts with 3 known pathogenic proteins including INS, KCNJ11, and GCK (Figure 3).

## 4. Discussion

In this study, we have investigated sequence variants in all genes in one patient with early-onset diabetes in a family with suspected MODY using WES. We did not identify rare nonsilent variants in known MODY genes. Two rare nonsilent variants in *EIF2AK3* and *GATA6* genes that are known to cause neonatal diabetes mellitus were classified as having pathogenic or likely pathogenic [15, 16], but the above two variants were not cosegregated with the disease. Only a rare heterozygous missense mutation in the last five candidate genes with predicted damaging effects in *IRS1* (rs1043152329) was cosegregated with diabetes, which is highly conserved across multiple species. In addition, *IRS1* acts together with known MODY pathogenic genes on the molecular pathway of T2DM and insulin signaling pathway and interacts with 3 known MODY pathogenic proteins; therefore, *IRS1* is probably the pathogenic gene of this family and rs1043152329 is the pathogenic mutation site.

IRS1 acts as a docking protein between the insulin receptor and multiple Src homology-2- (SH2-) containing proteins in the insulin signaling cascade, which plays a key role in modulating tissue response to insulin [17, 18]. *IRS1* has been considered a candidate gene for human metabolic disorders, especially in T2DM [19]. Mice deficient in *IRS1* by targeted disruption display insulin resistance and impaired glucose tolerance [20] and mice heterozygous for defects in genes for both *IRS1* and the insulin receptor or *IRS1* and glucokinase develop overt diabetes [21, 22]. To date, several genetic variants had been identified in human *IRS1*, which were associated with insulin resistance and diabetes susceptibility, particularly Gly(972)Arg (rs1801278) variant [23–29]. In addition, Zeggini et al. found that the Pro512Ala and Gly972Arg *IRS1* variants were associated with family history and early age of onset diabetes [30]. Previously studies reported that genetic variants (rs2943641; rs2943650) in *IRS1* were associated with insulin resistance, hyperinsulinemia, dyslipidemia, and T2DM [31–33]. Prudente et al. found that *IRS1* Gly972Arg polymorphism was associated with failure to oral antidiabetes drugs among patients with T2DM in a large and ethnically homogeneous sample [34]. Based on the above studies, which suggest the existence of an interaction between *IRS1* and diabetes, especially in T2DM, in this study, we found that *IRS1* is probably the pathogenic gene of this family; however, the functional mechanism by which *IRS1* p.His713Tyr variant contributes to diabetes remains unclear. Thus, we speculate that *IRS1* p.His713Tyr variant causes a defect in binding with PI3K, resulting in a decrease in IRS1-associated PI3K activity and subsequent activation of the kinase Akt and ultimately impairs the insulin message to the cellular vector pathways, thereby causing diabetes.

*GCK*, *HNF4A*, and *HNF1A* were the most frequently reported pathogenic genes causing MODY in Europeans, which accounts for approximately 94% of cases [35]. A previous result in MODY reported the prevalence of *HNF1A* mutations was 5% and the prevalence of *GCK* mutations was 2.5% in Korean [36]. The mutations of *HNF1A* (MODY3) and *GCK* (MODY2) accounted for 9% and 1% of MODY cases in China [10], respectively, and a majority of Chinese pathogenic gene of MODY is unknown. Further studies and increased sample size are required to estimate the pathogenic genes of MODY and their frequencies of early-onset diabetes patients in Asia.

Genetic testing is highly specific and sensitive and the gold standard for diagnosing MODY. Because of the cost, it is limited to use in all individuals. Whether genetic testing is applied in clinic depends on clinical manifestations, laboratory biochemical data, and patient's economic status. Shields et al. developed a clinical prediction model to determine the probability of MODY in patients with young-onset diabetes; they advised that their model should be used in all patients diagnosed with diabetes under the age of 35, who are not treated with insulin within 6 months of diagnosis; posttest probabilities of >25% would be appropriate to trigger genetic testing [8]. With the further reduction of the cost of genetic testing, its application in disease diagnosis will be more common and more mutations will be identified, which help us better distinguish between pathogenic mutations and rare mutations of no clinical significance. There were some limitations in this present study. First, about 90% of the sequences of all genes were covered by WES; therefore, about 10% of variants were missed. Second, it is difficult to classify the pathogenicity of novel rare variants. Third, the precise molecular mechanism underlying the influence of *IRS1* p.His713Tyr on the development of diabetes remains to be determined in further prospective studies.

In conclusion, WES could be an initial option for genetic testing in patients with early-onset diabetes. Our data suggest *IRS1* p.His713Tyr as a possible pathogenic mutation in monogenic diabetes, which might require further validation, and the precise molecular mechanism underlying the influence of *IRS1* p.His713Tyr on the development of diabetes remains to be determined in the further prospective studies.

## Figures and Tables

**Table 1:** Physical and laboratory examination.

	II-1	II-2	III-1	III-2	III-3	III-4	III-5	III-6	IV-1	IV-2
Gender	F	M	F	M	F	M	M	F	M	M
Age, y	77	71	38	36	58	60	57	54	30	28
Age of onset, y	64	48	—	—	—	45	32	—	—	—
BMI (kg/m^2^)	23.3	20.8	18.8	16.4	29.0	26.7	25.6	23.5	21.2	22.1
Urine sugar (neg)	Neg	Neg	Neg	Neg	Neg	4+	Neg	Neg	Neg	Neg
FPG (3.9∼6.1, mmol/L)	4.70	8.38	4.70	4.57	4.45	11.96	11.20	5.16	4.43	5.48
FCP (1.1∼3.3, ng/ml)	1.02	1.08	1.12	3.36	1.28	1.30	1.14	1.39	1.21	1.18
HbA1c (4∼6%)	5.3	8.6	5.3	5.4	5.3	10.2	9.0	5.6	5.3	5.3
Ketonuria (neg)	Neg	Neg	Neg	Neg	Neg	Neg	Neg	Neg	Neg	Neg
ALT (9∼50, U/L)	7.4	22.0	7.4	142.1	28.1	11.0	14.1	15.4	22.0	13.0
AST (15∼40, U/L)	12.5	18.6	12.5	41.7	24.8	13.0	17.1	21.2	25.3	13.5
Cr (57∼111, *μ*mol/L)	57.1	65.1	57.1	56.9	58.0	66.0	75.0	64.9	68.5	92.0
UA (208∼428, *μ*mol/L)	207.5	280.2	207.5	368.1	474.7	273.0	270.1	255.3	346.4	335.1
TG (<1.7, mmol/L)	0.41	0.87	0.41	1.34	1.34	1.22	1.16	1.53	1.78	0.61
TC (<5.18, mmol/L)	4.72	3.71	4.72	5.19	5.36	6.49	5.31	5.78	5.39	5.03
HDL-C (>1.04, mmol/L)	1.91	1.43	1.91	1.12	1.22	1.02	1.15	1.28	1.27	1.41
LDL-C (<3.37, mmol/L)	2.17	1.49	2.17	3.52	3.64	5.12	3.44	3.66	3.54	3.18
GAD (neg)	Neg	Neg	Neg	Neg	Neg	Neg	Neg	Neg	Neg	Neg
ICA (neg)	Neg	Neg	Neg	Neg	Neg	Neg	Neg	Neg	Neg	Neg
IAA (neg)	Neg	Neg	Neg	Neg	Neg	Neg	Neg	Neg	Neg	Neg

F: female; M: male; BMI: body mass index; FPG: fasting plasma glucose; FCP: fasting C-peptide; HbA1c: glycosylated hemoglobin; ALT: alanine aminotransferase; AST: aspartate aminotransferase. Cr: serum creatinine; UA: uric acid; TG: triglyceride; TC: total cholesterol; HDL-C: high-density lipoprotein cholesterol; LDL-C: low-density lipoprotein cholesterol; hsCRP: hypersensitive C reactive protein; GAD: glutamic acid decarboxylase antibodies; ICA: islet cell antibodies; IAA: insulin autoantibodies; Neg: negative.

**Table 2:** Whole-exome sequencing detail.

Exome capture statistics	Patient
Raw reads (bp)	209453466
Clean reads (bp)	209263948
GC (%)	51.93
Initial bases on target (bp)	60456963
Total effective reads (bp)	175646446
Total effective bases (Mb)	17512.80
Effective sequences on target (Mb)	12787.20
Capture specificity (%)	73.02
Mapping rate on genome (%)	99.92
Duplicate rate on genome (%)	16.07
Mismatch rate in target region (%)	0.32
Average sequence depth on target (X)	211.51
Fraction of target region covered ≥1x (%)	99.94
Fraction of target region covered ≥4x (%)	99.88
Gender	Male

**Table 3:** Information of candidate pathogenic gene loci.

CHR	POS	ID	REF	ALT	Gene	HGVSc	HGVSp
2	88890039		T	C	*EIF2AK3*	c.1087T > C	p.Ser363Pro
2	227661318	rs1043152329	G	A	*IRS1*	c.2137C > T	p.His713Tyr
10	101559103	rs149854486	C	T	*ABCC2*	c.1007C > T	p.Thr336Met
13	113765138		G	C	*F7*	c.265G > C	p.Glu89Gln
18	19757062		A	G	*GATA6*	c.1282A > G	p.Ile428Val

CHR: chromosome; POS: position; ID: identification; REF: reference; ALT: alternative; HGVSc: human genome variation society cDNA; HGVSp: human genome variation society protein.

**Table 4:** Pathogenicity of candidate gene mutation sites predicted by bioinformatics.

Gene	HGVSc	SIFT	PolyPhen2_HDIV	PolyPhen2_HVAR	MutationTaster	MutationAssessor	FATHMM	GERP_plus	PhyloP	PhastCons
*EIF2AK3*	c.1087T > C	0.082	0.001	0.002	1	1.355	−0.7	−2.75	−0.26	0
*IRS1*	c.2137C > T	0.022	0.576	0.123	0.949	1.1	0.42	3.87	1.241	0.887
*ABCC2*	c.1007C > T	0.504	0.031	0.025	1	0.7	−2.53	−6.28	−0.077	0
*F7*	c.265G > C	0.021	0.985	0.801	1	4.26	−6.94	4.34	5.64	1
*GATA6*	c.1282A > G	0.24	0.003	0.002	1	0.525	−6.31	4.11	7.229	1

HGVSc: human genome variation society cDNA; SIFT: deleterious (<0.05); PolyPhen2_HDIV: probably damaging (≥0.957), possibly damaging (0.453 ≤ pp2_hdiv ≤ 0.956); benign (≤0.452); PolyPhen2_HVAR: probably damaging (≥0.909), possibly damaging (0.447 ≤ pp2_hdiv ≤ 0.909); benign (≤0.446); MutationTaster: deleterious (>0.5); MutationAssessor: deleterious (>1.938); FATHMM: deleterious (<−1.5); GERP_plus: deleterious (>3); PhyloP: deleterious (>2.5); PhastCons: deleterious (>0.6).

**Table 5:** Sanger sequencing results of candidate gene loci in the family members.

Gene	HGVSc	HGVSp	Member of family	ALT	REF
*EIF2AK3*	c.1087T > C	p.Ser363Pro	II-1	AG	T
			II-2	AG	T
			III-1	AA	T
			III-2	AA	T
			III-3	AA	T
			III-4	AA	T
			III-5	AG	T
			III-6	AA	T
			IV-1	AA	T
			IV-2	AG	T
*IRS1*	c.2137C > T	p.His713Tyr	II-1	GA	C
			II-2	GA	C
			III-1	GG	C
			III-2	GG	C
			III-3	GG	C
			III-4	GA	C
			III-5	GA	C
			III-6	GG	C
			IV-1	GG	C
			IV-2	GG	C
*ABCC2*	c.1007C > T	p.Thr336Met	II-1	CC	C
			II-2	CC	C
			III-1	CC	C
			III-2	CC	C
			III-3	CC	C
			III-4	CC	C
			III-5	CT	C
			III-6	CC	C
			IV-1	CC	C
			IV-2	CC	C
*F7*	c.265G > C	p.Glu89Gln	II-1	GC	G
			II-2	GG	G
			III-1	GG	G
			III-2	GG	G
			III-3	GG	G
			III-4	GG	G
			III-5	GC	G
			III-6	GG	G
			IV-1	GG	G
			IV-2	GG	G
*GATA6*	c.1282A > G	p.Ile428Val	II-1	AG	A
			II-2	AG	A
			III-1	AA	A
			III-2	AA	A
			III-3	AA	A
			III-4	AA	A
			III-5	AG	A
			III-6	AA	A
			IV-1	AA	A
			IV-2	AG	A

HGVSc: human genome variation society cDNA; HGVSp: human genome variation society protein; ALT: alternative; REF: reference

**Table 6:** Evolutionary conservation analysis for the p.His713Tyr mutation in *IRS1*.

Protein acc.	Gene	Organism	Amino acid sequences		
NP_005535.1	*IRS1*	*H. sapiens*	697	KLWTNGVGGHHSHVLPHPKPPVESSGGKLLPCTGDYMNMSPVGDSNTSSP	746
XP_001134895.1	*IRS1*	*P. troglodytes*	696	KLWTNGVGGHHSHVLPHPKPPVESSGGKLLPCTGDYMNMSPVGDSNTSSP	745
XP_543274.3	*IRS1*	*C. lupus*	696	KLWTNGVGGHHPHALPHPKLPVESGSGKLLSCTGDYMNMSPVGDSNTSSP	745
XP_003585821.1	*IRS1*	*B. taurus*	697	KLWTNGVGGHHSHALPHPKLPVESGSGKLLSCTGDYMNMSPVGDSNTSSP	746
NP_034700.2	*Irs1*	*M. musculus*	692	KPWTNGVGGHHTHALPHAKPPVESGGGKLLPCTGDYMNMSPVGDSNTSSP	741
NP_037101.1	*IRS1*	*R. norvegicus*	692	KPWTNGVGGHHTHALPHAKPPVESGGGKLLPCTGDYMNMSPVGDSNTSSP	741
XP_004951158.1	*IRS1*	*G. gallus*	767	KIWTNGAG-----------------HHPKLSVESNEGKLPCGGGDYINMSPASGSTTSTP	809
XP_005157831.1	*IRS1*	*D. rerio*	631	KIWTNG------------------------INPKLSVESMEGKVSSC-GDYINMSPASCSTTSTP	670

**Table 7:** Analysis of molecular pathways for *IRS1* performed with KEGG.

Category	Term	*P* value	Genes
KEGG_pathyway	bta04930: type II diabetes mellitus	1.55*E* − 07	*GCK*, *INS*, *PDX1*, *IRS1*, *ABCC8*
KEGG_pathyway	bta04910: insulin signaling pathway	0.012273469	*GCK*, *INS*, *IRS1*
KEGG_pathyway	bta04960: aldosterone-regulated sodium reabsorption	0.055354558	*INS*, *IRS1*

## Data Availability

The data used to support the findings of this study are available from the corresponding author upon request.
